# Tumour Necrosis Factor-α Inhibition Improves Stroke Outcome in a Mouse Model of Rheumatoid Arthritis

**DOI:** 10.1038/s41598-019-38670-z

**Published:** 2019-02-18

**Authors:** N. R. Bonetti, C. Diaz-Cañestro, L. Liberale, M. Crucet, A. Akhmedov, M. Merlini, M. F. Reiner, S. Gobbato, S. Stivala, G. Kollias, F. Ruschitzka, T. F. Lüscher, J. H. Beer, G. G. Camici

**Affiliations:** 1 Center for Molecular Cardiology, University of Zurich, Schlieren, Switzerland; 2 First Clinic of Internal Medicine, Department of Internal Medicine, University of Genoa, Genoa, Italy; 3 Gladstone Institute of Neurological Disease; UCSF, San Francisco, CA USA; 4 Biomedical Sciences Research Center, Varkiza, Greece; 5 University Heart Center, University Hospital Zurich, Zurich, Switzerland; 6 Royal Brompton and Harefield Hospitals Trust, London, UK; 7 Department of Internal Medicine, Cantonal Hospital Baden, Baden, Switzerland

## Abstract

Rheumatoid Arthritis (RA) is a chronic inflammatory disorder where incidence and severity of myocardial infarction are increased. Data on the incidence and outcome of stroke are conflicting. Thus, we investigated outcome after Ischemia/Reperfusion (I/R) brain injury in a mouse model of RA and assessed for the role of the tumour necrosis factor-α (TNF-α) inhibitor Infliximab herein. We used a TNF-α reliant mouse model of RA. RA and wildtype (WT) animals were treated with vehicle (RA/WT) or Infliximab (RA Infliximab) for 4 weeks, before undergoing I/R brain injury. RA-animals displayed larger strokes and poorer neurological performance. Immunohistochemistry on brain sections revealed increased numbers of resident and peripheral innate immune cells (microglia and macrophages); increased Blood-Brain-Barrier (BBB)-disruption; decreased levels of the tight junction proteins (TJPs) claudin-5 and occludin; increased expression of matrix-metalloproteinases (MMP)-3 and -9 and enhanced lipid peroxidation. Treatment with Infliximab corrected these alterations. We show that RA associates to worse stroke-outcome via exacerbated BBB degradation by decrease of the TJPs claudin-5 and occludin. We identified MMPs-3 and -9 and increased oxidative stress as potential mediators thereof. Increased numbers of resident and peripheral innate immune cells (microglia and macrophages) may in turn contribute to all these effects. Infliximab-treatment restored the phenotype of RA-mice to baseline. Our data provide evidence clearly linking RA to adverse stroke-outcome in mice and indicate an approved TNF-α inhibitor as a potential strategy to reduce stroke-burden in this setting.

## Introduction

Stroke is the second-leading cause of death and the number one cause of permanent disability worldwide^1^, with acute ischemic stroke (AIS) accounting for 4 out of 5 cases.

AIS broadly affects many cerebral components, including the blood brain barrier (BBB) – a diffusion barrier consisting of endothelial cells, the basement membrane, pericytes and astrocyte end feet - which segregates the endovascular from the intra-parenchymal space and thereby protects the brain from frequent fluctuations in systemic homeostasis and invasion of peripheral immune cells^2^.

Inflammation is an important pathogenic component of AIS. Post-ischemically, it acts through a multicellular cascade involving both the adaptive and innate immune-systems at the local and systemic level^3^. Locally, the resident brain immune cells – microglia - undergo activation by damage associated molecular patterns (DAMPs) with consecutive secretion of pro-inflammatory cytokines. This in turn can facilitate the invasion of the ischemic brain by peripheral myeloid and lymphoid cells via BBB-degradation^4,5^.

Thus, patients suffering from a chronic inflammatory disease could at once experience a higher risk for and worsened outcome of stroke.

Rheumatoid arthritis (RA) is an immune-mediated, chronic inflammatory disorder. With a prevalence of ~1%, it ranks among the top 15% of diseases causing disability worldwide^6^. Apart from debilitating articular effects, associated systemic complications reduce median survival by 17 years^7^. Cardiovascular mortality is hereby increased by about 50%^8–11^. Particularly, the risk for myocardial infarction (MI) is increased by at least 2-fold and acute coronary syndromes in RA patients are clinically more severe and associate to higher fatality rates^12,13^. While the epidemiology of MI in RA is well characterized, the one of stroke is less defined with some studies reporting an increased risk^11,14–16^ and others finding no association^17–20^. Also, data on stroke outcome are conflicting, with some studies showing increased mortality rates and others not^7,10,21–26^. Meanwhile, data on clinical stroke severity and presentation are sparse.

TNF-α inhibitors, such as the monoclonal TNF-α antibody Infliximab, are clinically approved for the treatment of RA which remains active despite therapy with disease modifying anti-rheumatic drugs. TNF-α can play a dual role in stroke, promoting inflammatory stroke progression on one hand and mediating cerebral tolerance to hypoxia and ischemia on the other. Therefore, the potential effect of TNF inhibitors in RA patients with stroke is far from obvious^27^.

We hereby investigated outcome after I/R induced brain injury in a mouse model of RA and assessed for the role of Infliximab in this setting.

## Methods

### Animals

Sixteen weeks old male and female TNF-α transgene over-expressing mice on a CBA x C57BL/6 hybrid background were used as a murine model for RA^28^. Briefly, a 2.8 kb fragment of the human TNF-α genomic sequence comprising 0.6 kb of 5′ regulatory sequences, introns and exons up to the stop codon was used for the transgene. The 3′ region of the human TNF-α gene was replaced with that of the human β-globin gene, resulting in TNF-α dysregulation and pathology development^28^.

The RA mouse model exists in two severity degrees, depending on the copy number of the transgene. The more severely affected TG197 line expresses five copies, while the milder TG3647 line expresses only one^29,30^. At 6–8 weeks of age, the TG3647 line develops an arthritic phenotype with 100% penetrance. Symptoms chronically progress over a possible lifespan of about 1 year. Therefore, the line well-reflects chronic, adult-onset RA and allows for the study of advanced stages of RA. Anti-TNF-α treatment was shown to correct the arthritic phenotype of TG3647 mice^31,32^. To reduce post-surgical mortality and suffering, the milder TG3647 mouse line was used in thus study. The TG3647 mice will be referred to as RA-mice throughout the manuscript.

Breeding was maintained by crossing transgenic males with wild type (WT) females. Animals were maintained at 24 °C under a 12 h light/dark cycle and were fed a normal chow diet with ad libitum access to food and water. Study design and experimental protocols were approved by the institutional animal care committee (License N° TVA 077_2016; Kommission für Tierversuche des Kantons Zürich, Switzerland). All protocols were carried out in accordance with the relevant guidelines and regulations.

### Infliximab treatment

RA-mice of both sexes were randomly assigned to either weekly intraperitoneal injections of Infliximab (Remicade®, MSD AG, NJ, USA) at a clinically relevant dose (10 mg/kg bodyweight) for 4 weeks between weeks 12 to 16 (RA Infliximab), or an equivalent regimen of distilled water (RA). Wildtype animals received the same vehicle treatment (WT). Infliximab was acquired from the Zurich cantonal pharmacy.

### Clinical scoring

During the treatment period from week 12 to 16, animals were scored weekly using a clinical score for arthritic symptoms, social behaviour and bodyweight-loss.

The arthritic joint assessment was based on a previously published scoring system^33^: 0 (normal); 1 (edema or reddening of paw and/or ankle joints); 2 (distortion of paw and/or ankle joints); 3 (subluxation or ankyloses of paw and/or ankle joints).

Social behaviour was scored as follows: 0 (normal); 1 (minor changes, less interaction); 2 (burrowing or hiding, reduced mobility, isolation); 3 (no cage exploration, immobility). Finally, bodyweight was scored in this manner: 0 (steady weight or weight gain); 1 (weight loss of ≤5%); 2 (weight loss >5–10%); 3 (weight loss >10%).

Scores from each of the three categories were summed up to obtain the final score.

### Transient Middle Cerebral Artery Occlusion

Transient Middle Cerebral Artery Occlusion (tMCAO) was performed to induce I/R brain injury, as previously described^34,35^. Briefly, mice were anaesthetized using isoflurane 4% and 1.5% for induction and maintenance respectively. Body temperature was maintained at 37 °C. For analgesia, buprenorphine-HCL (0.1 mg/kg) was infiltrated subcutaneously at the incision site 30 minutes prior to surgery. The common, internal and external carotid arteries were dissected and a 6-0 silicone-coated filament (Doccol Corporation, Sharon, MA, USA) inserted into the common carotid artery and advanced to the origin of the left MCA to induce tMCAO for 45 min. After ischemia, middle cerebral artery (MCA) reperfusion was allowed for 24 h. During this time, animals were carefully observed and received analgesia with buprenorphine-HCL at a dose of 0.1 mg/kg s.c. every 6 hours. After 24 h of reperfusion, mice were sacrificed using an overdose of CO_2_.

### Stroke Volume

After euthanasia, mice were perfused with 10 ml of phosphate buffered saline (PBS) and relevant organs were excised. Murine brains were cut into 6 equally spaced (1 mm) coronal sections and immersed in a 2% solution of 2,3,5-triphenyltetrazolium chloride (TTC) (Sigma-Aldrich, Chemie GmbH, Buchs, Switzerland) at 37 °C for 20 min. TTC is a redox indicator, which changes its colour from white to red, as it gets reduced to 1,3,5-triphenylformazan in viable tissues. In metabolically inactive tissues lacking cellular respiration, this reduction does not take place, leaving TTC in its unreacted state. Therefore, the stroke area can be distinguished from viable brain tissue by its white appearance^36^.

Areas of infarction, ipsilateral and contralateral hemispheres were quantified using ImageJ software (Image J, NIH, MD, USA). To correct infarct size measurement for cerebral edema and consequent overestimation, we applied the following formula as previously described^37^: Corrected infarct volume = contralateral hemisphere volume - (ipsilateral hemisphere volume - infarct volume). Infarct size was expressed as volume in mm^3^.

### Neurological Deficiency Assessment

Baseline and post-infarction neurological status were assessed by a four-point scale neurological score according to Bederson *et al*.^38^ and the RotaRod test as previously described^34^. Both tests are composite sensory-motor tests, which take into account motor functions, proprioception, spatial orientation and balance^39^. The neurological score test according to Bederson was performed at baseline, 2 h and 24 h after reperfusion and consists of the following scores: grade 0, normal neurological function; grade 1, forelimb and torso flexion on and towards the contralateral side upon lifting of the animal by the tail to 1 m above the work surface; grade 2, circling to the contralateral side; grade 3, leaning to the contralateral side at rest; grade 4, no spontaneous motor activity. The RotaRod test was performed at baseline and 24 h after reperfusion. To this purpose, mice were placed on a rotating rod at increasing speed and the time to tendency to fall was measured in seconds. Per animal, three consecutive measurements were performed at each time-point and the best score was used.

### Histology

Histological procedures were performed as previously described^40,41^. Briefly, following euthanasia, mice were exsanguinated by cardiac puncture and transcardially perfused with PBS and 4.0% paraformaldehyde (PFA) (Sigma-Aldrich, Chemie GmbH, Buchs, Switzerland) in PBS at room temperature. The brains were removed and consecutively incubated overnight in 4.0% PFA at 4 °C and afterwards transferred to 30% sucrose in PBS for 36 h. Cryoprotected brains were cut into 100-µm thick free-floating sections using a microtome (Leica Jung HN40), pre-treated with proteinase K or 1 M HCL for antigen retrieval and immune-blocked with 10% donkey serum. After these steps, they were incubated with primary antibodies at the following dilutions: Iba-1 (1:500; Wako Chemicals, Osaka, Japan), Occludin (1:200; Santa Cruz Biotechnology, Santa Cruz, CA, USA), the endothelial marker CD31 (1:50; BD Pharmingen, Allschwil, Switzerland), claudin-5 (1:200; Abcam, Cambridge, United Kingdom), VE-cadherin (1:200; Abcam, Cambridge, United Kingdom), 4-Hydroxynonenal (4-HNE) (1:200; Abcam, Cambridge, United Kingdom), MMP-3 (1:100; Abcam, Cambridge, United Kingdom) and MMP-9 (1:500; Abcam, Cambridge, United Kingdom) at 4 °C overnight, respectively. Secondary antibodies were added at a dilution of 1:750 (Jackson Immunoresearch, West Grove, PA, USA) for 24 h at 4 °C. Images were acquired using a confocal microscope (Leica SP8; Leica, Wetzlar, Germany). Cells positively stained for the microglial and activated macrophage marker Iba-1, were counted in a specified ipsilateral area (the CA1 hippocampal area) using ImageJ software. Stained areas of claudin-5, occludin, VE-cadherin, MMP-3 and MMP-9 were measured using ImageJ software (Image J, NIH, MD, USA) and normalized to the total endothelial cell surface area assessed by CD31 staining. The area positively stained for 4-HNE on the ipsilateral hemisphere was measured using ImageJ software (Image J, NIH, MD, USA) and expressed as a percentage of the contralateral hemisphere.

BBB permeability was assessed by quantifying endogenous immunoglobulin G (IgG) extravasation. Sections were incubated with Alexa647-conjugated donkey anti-mouse IgG for 24 h (1:600; Jackson Immunoresearch, West Grove, USA). IgG-stained area was expressed as a percentage of the contralateral hemisphere.

### Statistical Analysis

Data were expressed as mean ± standard error of the mean (SEM). All statistical analyses were performed using GraphPad Prism 7 software (GraphPad Software, Inc, La Jolla, CA, USA). Results were confirmed to follow a normal distribution with the Kolmogorov-Smirnov test of normality. Data that passed the normality assumption were analyzed with two-tailed unpaired Student’s t-test and data that failed the normality assumption were analyzed with the nonparametric Mann–Whitney *U* test. For repeated measurements, two-way ANOVA with Sidak *post hoc* test was applied. A probability value (P) below 0.05 was considered statistically significant. For the graphical depiction of p-values in the figures, the following applies: *P < 0.05, **P < 0.01, ***P < 0.001.

## Results

### RA mice develop a progressive arthritic phenotype which can be averted by Infliximab

**Figure 1:**
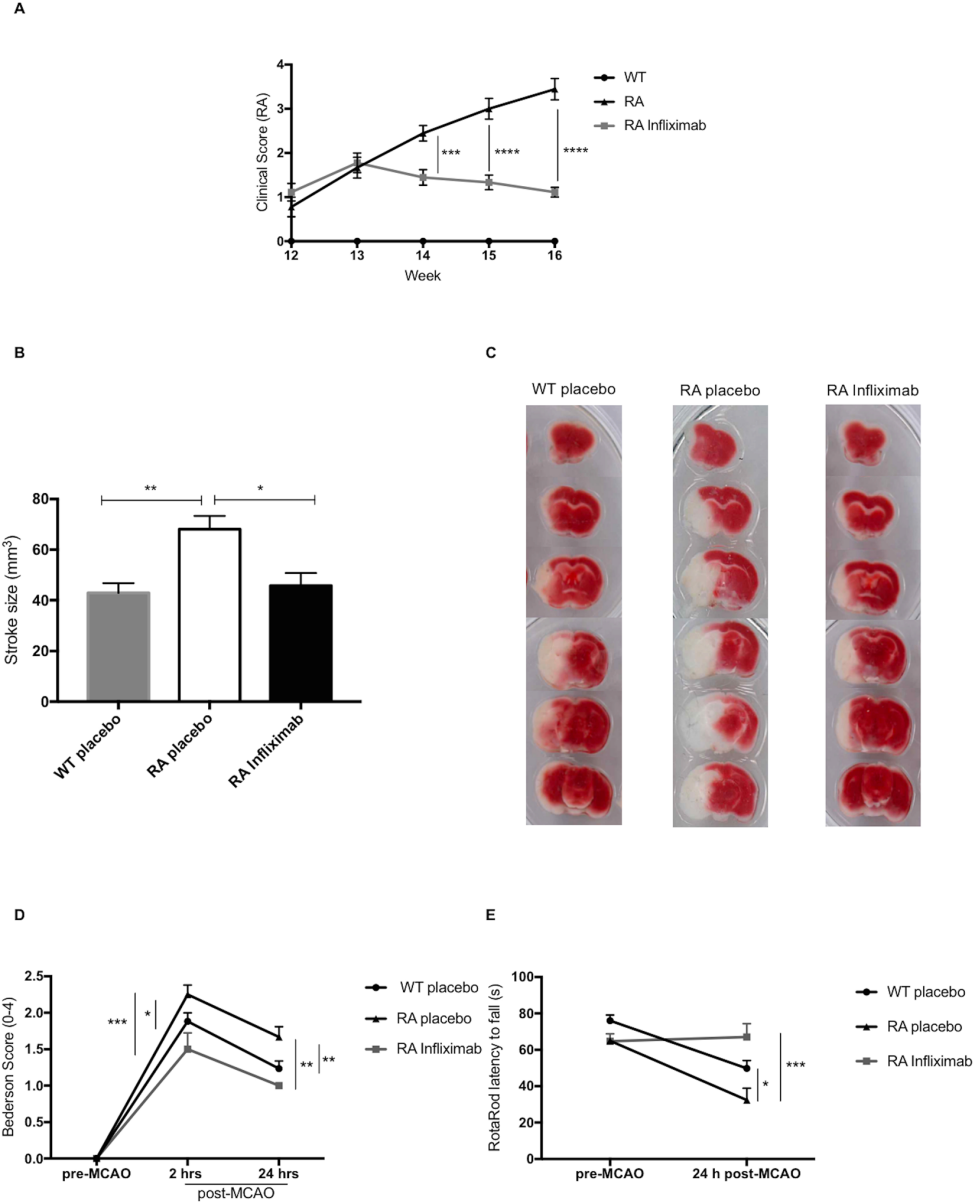
Arthritic phenotype and morphological and functional stroke outcome in RA mice: (**A**) Clinical score of WT, RA and Infliximab-treated RA mice during the treatment period between weeks 12 and 16. (**B**) Brain infarct volume 24 h after tMCAO was measured on TTC stained coronal sections of WT, RA and Infliximab-treated RA mice, whereby the stroke area is distinguishable from viable tissue by its white colour (**C**). (**D**) Neurological deficit assessed by a Bederson-based score of 0–4 in WT, RA and Infliximab-treated RA animals. (**E**) Neurological function assessed by RotaRod and expressed as latency to fall in WT, RA and Infliximab-treated RA animals.

RA mice displayed a progressive increase in the composite clinical score over time. At the age of 12 weeks, RA animals of both the treatment and the control groups showed relatively mild arthritic symptoms. Thereafter, symptom severity steadily increased in the vehicle-injected animals, while Infliximab treatment could blunt the increase (week 14: RA 2.44 ± 0.53, n = 9 vs. RA Infliximab 1.44 ± 0.52, n = 9; p < 0.0001; week 15: RA 3 ± 0.70, n = 9 vs. RA Infliximab 1.33 ± 0.50, n = 9; p < 0.0001; week 16: RA 3.44 ± 0.72, n = 9 vs. RA Infliximab 1.11 ± 0.33, n = 9; p < 0.0001; Fig. 1A). At all time-points, treated and untreated RA animals scored significantly higher than WT animals, where the clinical score remained 0 for the whole duration of the study.

### RA mice display larger strokes and worse neurological function upon I/R brain injury

Upon tMCAO, we observed significantly larger stroke volumes in RA compared to WT animals (WT: 42,88 ± 3,87 mm^3^, n = 12 vs. RA: 68,1 ± 5,26 mm^3^, n = 6; P = 0.0024; Fig. 1B,C).

Weekly treatment with Infliximab over 4 weeks, restored stroke volumes in RA animals to levels observed in WTs (RA: 68,1 ± 5,26 mm^3^, n = 6 vs. RA Infliximab: 45,8 ± 4,99 mm^3^, n = 6; P = 0.0192; WT vs. RA Infliximab; P = NS; Fig. 1B,C).

Neurological performance was significantly impaired in RA compared to WT animals, as assessed by the Bederson test (2 h: WT 1.88 ± 0.12, n = 17 vs. RA 2.25 ± 0.13, n = 12; P = 0.0270; 24 h: WT 1.24 ± 0.10, n = 17 vs. RA 1.67 ± 0.14, n = 12; P = 0.0075) and the RotaRod test (WT 49.82 ± 4.4 s, n = 17 vs. RA 32.3 ± 6.5 s, n = 12; P = 0.0128). Infliximab-treatment improved neurological function in the Bederson (2 h: RA 2.25 ± 0.13, n = 12 vs. RA Infliximab 1.5 ± 0.22, n = 6; P = 0.0003; 24 h: RA 1.67 ± 0.14, n = 12 vs. RA Infliximab 1 ± 0, n = 6; P = 0.0015; WT vs. RA Infliximab (all time points); P = NS; Fig. 1D) as well as the RotaRod test (RA: 32.3 ± 6.5 s, n = 12 vs. RA Infliximab: 67 ± 7.4 s, n = 6; P = 0.0001; WT vs. RA Infliximab; P = NS; Fig. 1E). On a more descriptive note, 2 h after reperfusion, all WT and Infliximab-treated mice had a Bederson score of either 1 or 2, which corresponds to forelimb flexion on the contralateral side or circling to the contralateral side respectively. Meanwhile, 25% of the RA animals scored 3 at 2 h, which corresponds to leaning to the contralateral side even at rest. None of the RA animals displayed only mild neurological symptoms (Bederson score 1) at any time point.

At 24 h post reperfusion, the majority of WT and all of the RA Infliximab animals retained only mild neurological symptoms (Bederson score 1), while 67% of RA animals still displayed circling to the contralateral side (Bederson score 2).

### RA mice display increased numbers of microglia and activated macrophages

Microglia as the resident immune cells of the brain, are the first to respond to exposure of damage associated molecular patterns (DAMPs) following ischemia. Upon activation, they can secrete pro-inflammatory cytokines such as TNF-α and IL-1β and thereby exacerbate neuronal injury and para-cellular permeability.

Ionized calcium binding adaptor molecule 1 (Iba-1) is a marker for both microglia and activated macrophages. In order to assess the contribution of microglia and invading macrophages (M/M) to the observed stroke phenotype, we have measured Iba-1 as a surrogate for these cells in the ipsilateral hemisphere.

**Figure 2:**
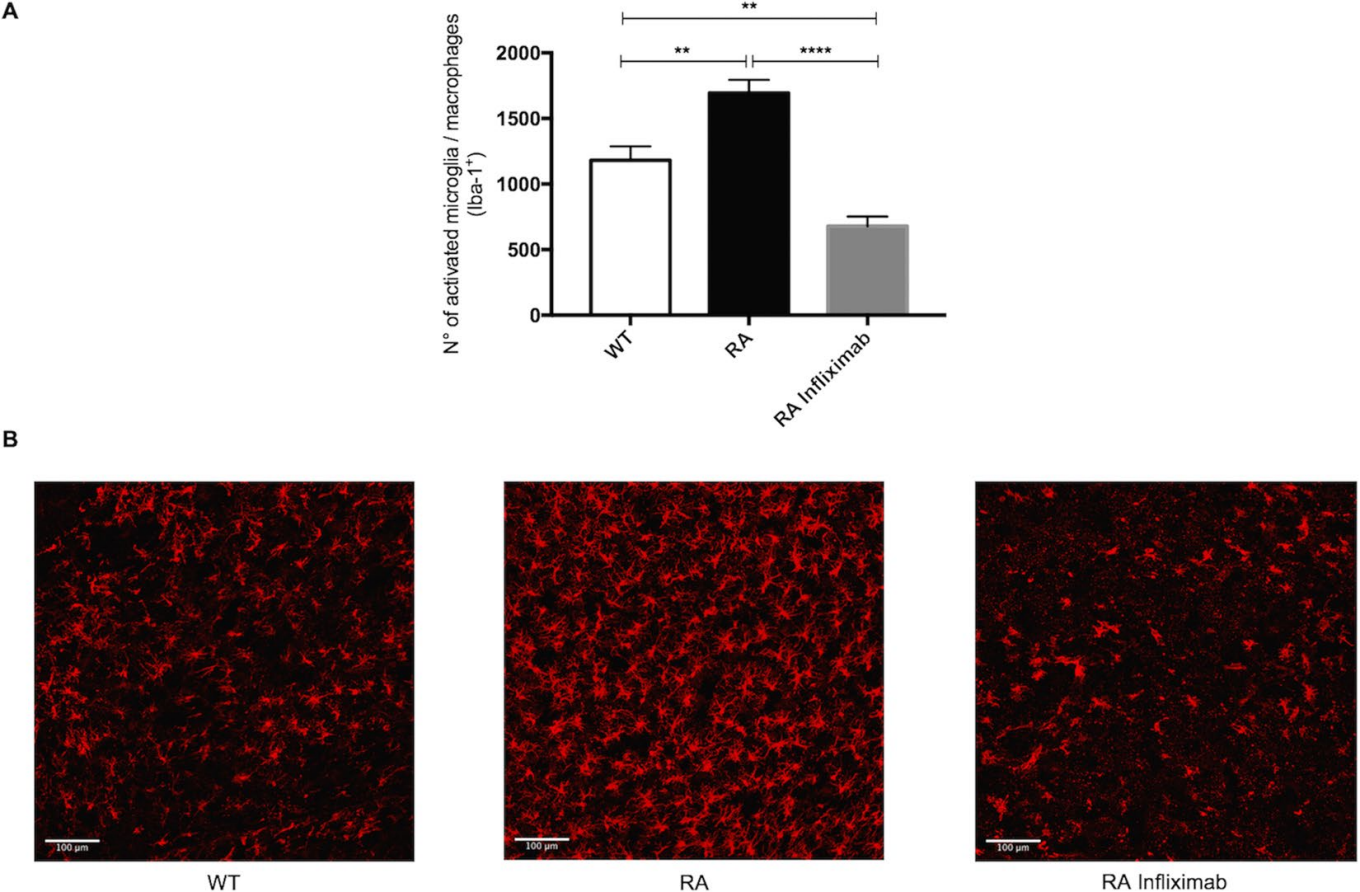
RA associates to increased post-ischemic counts of microglia and activated macrophages. (**A**) Numbers of microglia and activated macrophages as determined by Iba-1 staining in a specified area of the stroke hemisphere 24 h after tMCAO in WT, RA and RA Infliximab mice. (**B**) Representative images showing microglia and activated macrophages (red) in the stroke hemispheres of WT, RA and RA Infliximab mice 24 h after tMCAO.

RA animals showed significantly increased numbers of M/M in the stroke area compared to WT mice (WT: 1180 ± 303, n = 8 vs. RA: 1694 ± 280, n = 8; P = 0.0026). Infliximab treatment meanwhile significantly lowered the count of these resident and peripheral immune cells compared to both untreated RA animals and even WT mice (RA: 1694 ± 280, n = 8 vs. RA Infliximab 678 ± 208, n = 8; P < 0.0001; WT: 1180 ± 303, n = 8 vs RA Infliximab 678 ± 208, n = 8; P = 0.0032, Fig. 2A,B).

### RA mice display increased BBB disruption and tight junction protein (TJP) degradation

I/R-induced BBB breakdown is characterized by extravasation of large circulating molecules, such as IgG into the affected parenchyma^42^. A disrupted BBB allows for the formation of vasogenic edema and the invasion of systemic inflammatory cells, which both drive stroke progression. It is thus an important mediator of unfavourable stroke outcome. As to be expected, we observed some IgG extravasation into ischemic hemispheres in all experimental groups, whereas the contralateral hemispheres remained unaffected.

**Figure 3:**
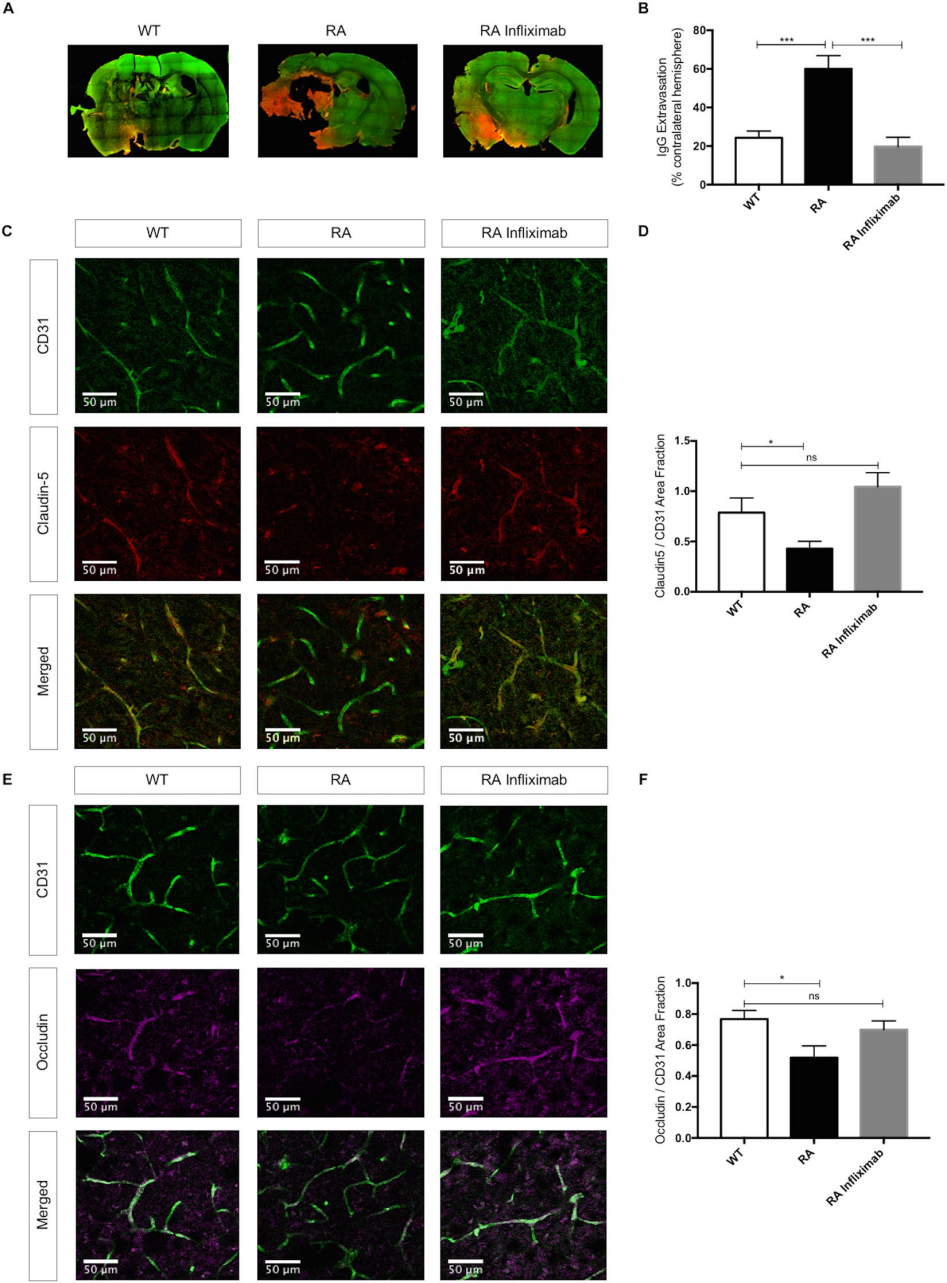
RA associates to increased blood brain barrier permeability through decreased tight junction protein levels: (**A**) Representative images showing endogenous IgG extravasation (red) into the brain parenchyma (green for CD31) 24 h after tMCAO in WT, RA and RA Infliximab mice. (**B**) Endogenous IgG extravasation 24 h after tMCAO expressed as a percentage of the contralateral hemisphere. (**C**,**E**) Representative immunostainings for claudin-5 (red) and occludin (magenta) and CD31 (green) in ipsilateral hemispheres 24 h after tMCAO. (**D**,**F**) Quantification of claudin-5 and occludin positive areas normalized to the total endothelial surface area (CD31) in ipsilateral hemispheres of WT, RA and RA Infliximab animals.

RA mice displayed aggravated BBB disruption upon I/R, as documented by increased extravasation of IgG (WT: 24,33 ± 3,46%, n = 8 vs. RA: 60,06 ± 6,87% n = 6; P = 0.0002). Infliximab-treatment blunted IgG extravasation to baseline levels (RA: 60,06 ± 6,87%, n = 6 vs. RA Infliximab: 19,72 ± 4,82%, n = 8; P < 0.0001; WT vs. RA Infliximab; P = NS, Fig. 3A,B).

Integrity of the endothelial component of the BBB is regulated by tight and adherens-junctional proteins (TJP). During stroke, paracellular permeability increases due to the degradation of these proteins. Here, we assessed the integrity of the 2 TJP occludin and claudin-5 as well as the adherens junction protein VE-cadherin by immunostaining.

Both claudin-5 and occludin tissue protein levels were lower in the ipsilateral hemispheres of RA compared to WT-animals (claudin-5; WT: 0,79 ± 0,15, n = 8 vs. RA: 0,43 ± 0,07, n = 10; P = 0.0328; Fig. 3C,D/occludin; WT: 0,77 ± 0,06, n = 6 vs. RA: 0,52 ± 0,08, n = 8; P = 0.0284; Fig. 3E,F**)**. VE-cadherin expression was unaltered. The representative images depict a specified area in the ipsilateral hemisphere, where the vasculature appears in green in the top panel (for the endothelial marker CD31) and the respective TJPs in red or magenta in the middle panel. The bottom panel shows an overlay of the TJPs with the total endothelial surface, where a reduction of said proteins in the RA animals becomes apparent.

Infliximab-treatment of RA animals restored the expression of claudin-5 (WT: 0,79 ± 0,15, n = 8 vs. RA Infliximab: 1,04 ± 0,14, n = 11; P = NS, Fig. 3C,D) and occludin (WT: 0,77 ± 0,06, n = 6 vs. RA Infliximab: 0,70 ± 0,06, n = 10; P = NS, Fig. 3E,F) to WT levels. Moreover, treatment with Infliximab significantly increased claudin-5 expression compared to vehicle treated RA animals (RA: 0,43 ± 0,07, n = 10 vs. RA Infliximab: 1,04 ± 0,14, n = 11; P = 0.001).

### RA mice display higher levels of MMP-3 and MMP-9 and increased lipid peroxidation

**Figure 4:**
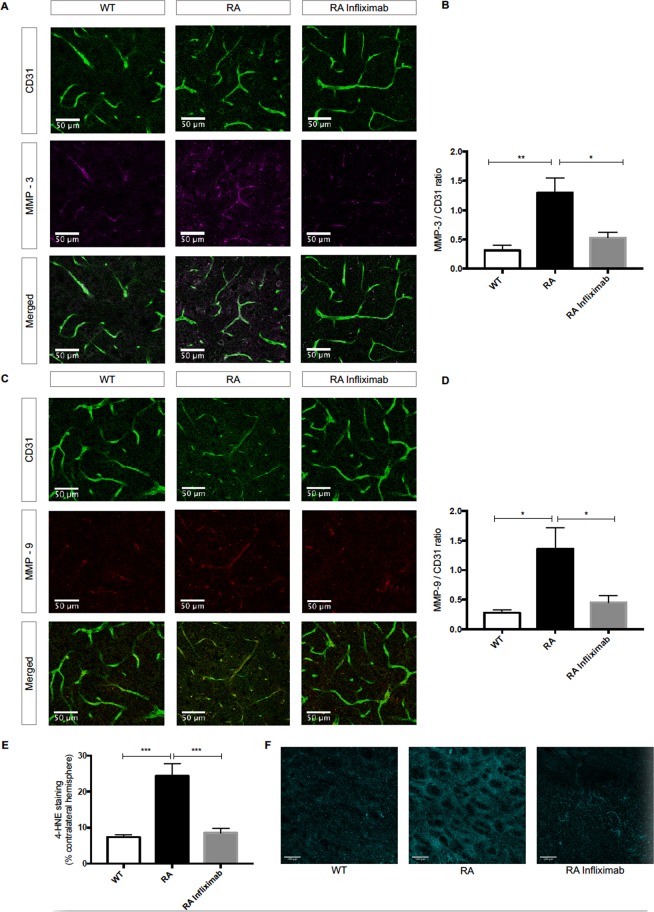
RA associates to increased post-ischemic levels of MMP-3 and -9 and oxidative stress. (**A**,**C**) Representative immunostainings for MMP-3 (magenta) and MMP-9 (red) and CD31 (green) in ipsilateral hemispheres of WT, RA and RA Infliximab animals, 24 h after tMCAO. (**B**,**D**) Quantification of MMP-3 and MMP-9 positive areas normalized to total endothelial surface (CD31 positive area) in ipsilateral hemispheres. (**E**) Quantification of ipsilateral area positively stained for 4-HNE expressed as percentage of the contralateral hemisphere in WT, RA and RA Infliximab animals. (**F**) Representative images of immunoreactivity of 4-HNE (cyan) in the stroke area of WT, RA and RA Infliximab animals 24 h after tMCAO.

As potential mediators of increased TJP-degradation, expression levels of MMPs-3 and −9 were evaluated by immunostaining. We found augmented expression of MMP-3 in RA compared to WT animals (WT: 0.31 ± 0.09, n = 6 vs. RA: 1.30 ± 0.25, n = 7; P = 0.0023, Fig. 4A,B). MMP-9 expression levels were also raised in RA compared to WT mice (WT: 0.28 ± 0.05, n = 6 vs. RA: 1.36 ± 0.35, n = 7; P = 0.0106, Fig. 4C,D). Infliximab-treatment blunted MMP-3 (RA: 1.30 ± 0.25, n = 7 vs. RA Infliximab: 0.53 ± 0.09, n = 7; P = 0.0116, Fig. 4A,B) and MMP-9 (RA: 1.36 ± 0.35, n = 7 vs. RA Infliximab: 0.45 ± 0.11, n = 7; P = 0.0254, Fig. 4C,D) levels. The representative images depict a specified area in the ipsilateral hemisphere, where the vasculature appears in green in the top panel (for the endothelial marker CD31) and the respective MMPs in red or magenta in the middle panel. The bottom panel shows an overlay of MMP immunoreactivity with the total endothelial surface, where an increase of said enzymes in the RA animals becomes apparent.

Increased oxidative stress is known to activate MMP-3 and MMP-9^43^. Thus, we assessed for post-ischemic oxidative stress levels by immunostaining for 4-HNE – a marker of lipid peroxidation.

4-HNE levels were increased in RA compared to WT mice (WT: 7.38 ± 0.67%, n = 9 vs. RA: 24.37 ± 3.45%, n = 9; P < 0.0001; Fig. 4E,F). Infliximab-treatment of RA-mice blunted lipid peroxidation (RA: 24.37 ± 3.45%, n = 9 vs. RA Infliximab: 8.53 ± 1.24%, n = 6; P = 0.0005; Fig. 4E,F). The representative images show 4-HNE immunoreactivity (cyan) in the stroke area of WT, RA and RA Infliximab animals.

## Discussion

RA is a chronic systemic, immune-mediated inflammatory disorder. Apart from debilitating articular effects, RA associates to a decreased median survival^7^, mainly driven by cardiovascular complications. In particular, the frequency, clinical severity and mortality of acute coronary syndromes are increased^12,13^. Data on incidence and mortality of stroke in RA patients are more conflicting^16,19^, while data on clinical severity are sparse. Moreover, the influence of biologicals like Infliximab on stroke outcome in RA patients remains unclear^27^. Filling this knowledge gap is crucial for designing personalised therapeutic approaches to improve prognosis in these patients. To this end, we aimed to investigate the morphological and functional outcome after I/R brain injury in a RA mouse model.

RA-mice displayed an increased stroke volume by approximately 60% compared to WTs. The physiological relevance of these findings was supported by a greater post-ischemic neurological impairment in RA animals. Infliximab-treatment of RA mice not only improved arthritic symptoms, but also reduced stroke volumes and neurological dysfunction to WT levels, thereby confirming the relevance of inflammation and in particular, of the cytokine TNF-α.

Microglia are the resident brain immune cells and as such, the first line of response to damage associated molecular patterns exposed upon ischemia^3^. In previous studies, they have been shown to be important secretors of pro-inflammatory cytokines such as TNF-α and IL-1β in the brain^44^. Furthermore, microglia-derived TNF-α was shown to induce apoptosis in neuronal precursor cells^45^, attract peripheral immune cells^46^ and facilitate extravasation of immune cells, platelets, erythrocytes and fibrin by increasing endothelial adhesion molecule expression^47,48^. Moreover, in an attempt at neo-vascularization upon ischemia, microglia along with endothelial cells and astrocytes increase the generation of matrix metalloproteinases, which in turn contribute to BBB dysfunction by degrading extracellular matrix and tight junction proteins^49^.

We found increased numbers of activated microglia and macrophages in the stroke hemispheres of RA animals, when compared to WT. Treatment with Infliximab meanwhile significantly decreased the post-ischemic counts of these cells, both compared to untreated RA animals and WT mice. These findings suggest not only a pertinent role of resident and peripheral immune-cells in mediating the observed stroke phenotype, but also a role for TNF-α in activating and recruiting these cells to the site of injury.

BBB disruption is a hallmark of stroke and a mediator of brain injury and stroke progression^50^. This notion is supported by data showing BBB-breakdown as early as 30 minutes after ischemia^51^. Inflammatory cytokines play a crucial role in this respect. Indeed, TNF-α and IL-6 were shown to dose-dependently decrease tight- and adherens-junction expression *in vitro*^52^ and to be increased in ischemic hemispheres of rats after tMCAO^53^. Furthermore, TNF-α serum- and cerebrospinal fluid-levels are increased in stroke patients and positively correlate to stroke severity^54^.

We found that RA mice display an increased BBB disruption after stroke as compared to WTs. As possible mediators thereof, we describe reduced protein levels of the TJPs claudin-5 and occludin. These transmembrane proteins are the backbone of cerebral endothelial TJs, which uphold three key functions: blocking the paracellular diffusion of blood-borne polar substances; maintenance of cell polarization by preventing lateral diffusion of lipids and membrane proteins and the provision of an intracellular signalling platform. Generally, distancing of these proteins from the cellular borders or decreases in their expression at the TJ cleft mediate a loss of junctional integrity and increased paracellular permeability, which in turn directly contributes to vasogenic edema, haemorrhagic transformation and increased mortality^55,56^. Infliximab-treatment of RA mice restored TJP expression to WT levels and in doing so, also conserved BBB integrity as demonstrated by diminished IgG extravasation.

MMPs are proteolytic enzymes degrading all components of the extracellular matrix. In cerebral ischemia, they can mediate early degradation of TJPs^50,57^. The main inducible MMPs in the brain are MMP-3 and MMP-9^58^. Thus, we investigated their putative involvement in the observed BBB-breakdown and found that expression levels of the active enzymes were significantly increased in ischemic hemispheres of RA animals and that these alterations could be reversed by Infliximab, supporting the crucial role of inflammation in post-ischemic MMP expression.

Both MMPs-3 and -9 are secreted in a latent form to undergo activation by nitrosylation or oxidation^59^, whereas active MMP-3 can also directly activate MMP-9. Additionally, oxidative stress has permeabilizing actions of its own and is subject to inflammatory stimuli. Thus, we investigated oxidative stress levels and showed that ipsilateral hemispheres of RA animals present significantly more lipid peroxidation – a marker of oxidative stress - as compared to WT, while Infliximab-treatment blunted this increase. This indicates that oxidative stress partially links inflammation to BBB disruption in our model.

The overall-beneficial effects of Infliximab not only underline the pertinence of inflammation to the observed changes in RA mice, but also implicate this drug as a potential modulator of stroke in RA-patients.

One limitation of the herein reported study is represented by the use of vehicle as a control to Infliximab as opposed to using an isotype control antibody. IgG are capable of regulating the immune response through Fc binding alone^60^ thus, the optimal control would have to be an isotype antibody. However, the key role of TNF-α in the pathogenesis of RA is understood^61–63^; accordingly, anti-TNF-α treatment is well-established and beneficial in RA patients^64^. Taken together, this should confer additional confidence that the observed effects in our RA mouse model are specific to the antibody used.

In conclusion, results from this study contribute additional mechanistic evidence to epidemiological data concerning the link between RA and stroke. They call for further clinical studies in this direction to allow for the design of personalised treatment strategies, which could involve tailored use of biologicals such as Infliximab.
